# Supplementary material for: A GIS-based policy support tool to determine national responsibilities and priorities for biodiversity conservation
Source: PLoS One. 2020 Dec 3;15(12):e0243135. doi: 10.1371/journal.pone.0243135 (PMC7714368; doi:10.1371/journal.pone.0243135)
Supplement: S1 File — (PDF) [file pone.0243135.s001.pdf]

Supporting Information S1 File: NRA-tool user interface

The first step in operating the NRA-Tool is to open the biogeographic units, reference region, focal species, and focal region layers/shapefiles (Fig S1.1, Fig S1.2). After clicking the "Layers" tab, the focal species should be selected in the "Species layers" text box. Biogeographic units and the corresponding field names should be selected by clicking the drop-down arrows "Biome layer" and "'Name' field for biomes" (Fig S1.2a). A reference region layer and the corresponding field name must be selected by clicking the drop-down arrows "Reference region layer", and "'Name' field for ref. regions" (e.g., the Global Environmental Zone layer with name field "GENZNAME"). Similarly, a focal region layer and the name of the corresponding field must be selected by clicking the drop-down arrows "Focal region layer" and "'Name' field for foc. regions" (e.g., the focal region layer with field name "CNTRY_NAME"). By clicking on "Selected values" and choosing focal regions, users can select particular regions or countries from the "Selected values" list box. With the tab "Filter layers" the species, reference region shapefiles and focal region shapefiles can be selected from the list of shapefiles in the directory by filtering the prefix of the files. Filtering by s-*name* results in species shapefiles, by g-*name* results in reference region shapefiles, and by f-*name* results in focal region shapefiles.

Click the "Parameters" tab of the NRA-Tool window (Fig S1.2b) for setting the calculation type; the distribution parameters, such as determinants of either the area or the number of biophysical regions; a distribution factor (e.g., ratio of DP_{exp} and DP_{obs}); an International Union for Conservation of Nature (IUCN) category file including "Species Scientific Name", "Species English Name", and "IUCN Level"; and an output file, such as a shapefile that stores the geometric location. NR attribute information of geographic features based on the above DP_{exp} and DP_{obs} results for calculating NR and CP values should be defined before the "Analyze" button is clicked. The number of biogeographic regions covered by species or distribution sizes of species must be selected by clicking on the "Local", "Regional", and "Wide" drop-down arrows or by directly entering values in the corresponding text boxes. A distribution coefficient (i.e., ratio) must be selected by clicking the "Distribution coef." drop-down arrow or directly by entering values in the corresponding box. A calculation type for the NR analysis can be selected by clicking the "Calculation type" drop-down arrow and choosing either the Polygon-Count Approach or the Polygon Area-Approach.

In addition, a list file with IUCN names in comma separated value (CSV) format should be selected by clicking the "IUCN category" button to navigate to a folder containing the

IUCN names list file, or by simply typing the name of the folder containing the IUCN list file in the corresponding text box. If the layer name of the focal species does not exactly match the English name in the IUCN category file, a dialog box as shown in Fig S1.2c is generated to select and confirm the name of the focal species from the IUCN category file (see Table S1.1).

Table S1.1: Example of how the ASCII text file looks in a text editor program. The first line contains the field names, the following lines contain the data. Fields are separated by semicolons.

`Species_Scientific_Name;Species_English_Name;IUCN_Level`
`Pyrrhoplectes epauletta;Gold-naped Finch;LC`
`Pyrrhula erythaca;Gray-headed Bullfinch;LC`
`...;...;...`
`Corvus torquatus;Collared Crow;NT`
`Sitta magna;Giant Nuthatch;VU`
`...;...;...`
`Sitta europaea;Eurasian Nuthatch;LC`

To add thematic layers such as CN and CP layers, select the option “Add thematic layers” (Fig S1.2b). NR and CP results are saved either by clicking the “Save results to” button to navigate to a working folder and output file name, or by typing the output shapefile name directly in the corresponding text box. Previously obtained results can be overwritten by new results by selecting “Overwrite the results” option (Fig S1.2b).

The final step is to click the “Analyze” button to run the NRA-Tool to calculate both NR and CP. Fig S1.2a shows the processing bar that pops up when all settings have been made correctly. If multiple species are selected as focal species, users are prompted to select the results that will be visually displayed on the NR and CP maps on the NRA-Tool ArcGIS platform (Fig S1.4a and d).

The results of the analyses can be browsed by clicking the “Results” tab (Fig S1.2b). The output shapefile with its attributes table is automatically saved in the predefined working folder. After the NR and CP analysis, the “Creating thematic layers”-dialog box is displayed to create NR and CP maps (Fig S1.2a and c, left-hand side of windows as in Fig S1.4b and c). To view NR and CP results and maps obtained with different calculation types in all runs, click the “Add layers from results” button to display the “Creating thematic layer” dialog box. However, the NRA-Tool also provides the option to perform further analysis based on the NR and CP results (Fig S1.4d). For example, to determine the number of NR levels and CP classes for each region, click the “Analyze NR and CP results” button (Fig S1.2a).

Fig S1.1. The National Responsibility Tool interface.
Panel (a) shows the Global Environmental Zones (GEnZ, [1]) while panel (b) shows the Terrestrial Ecoregions of the World (TErW, [2]). In both, the distribution area of the Fairy Pitta (Pitta nympha) is outlined in green.

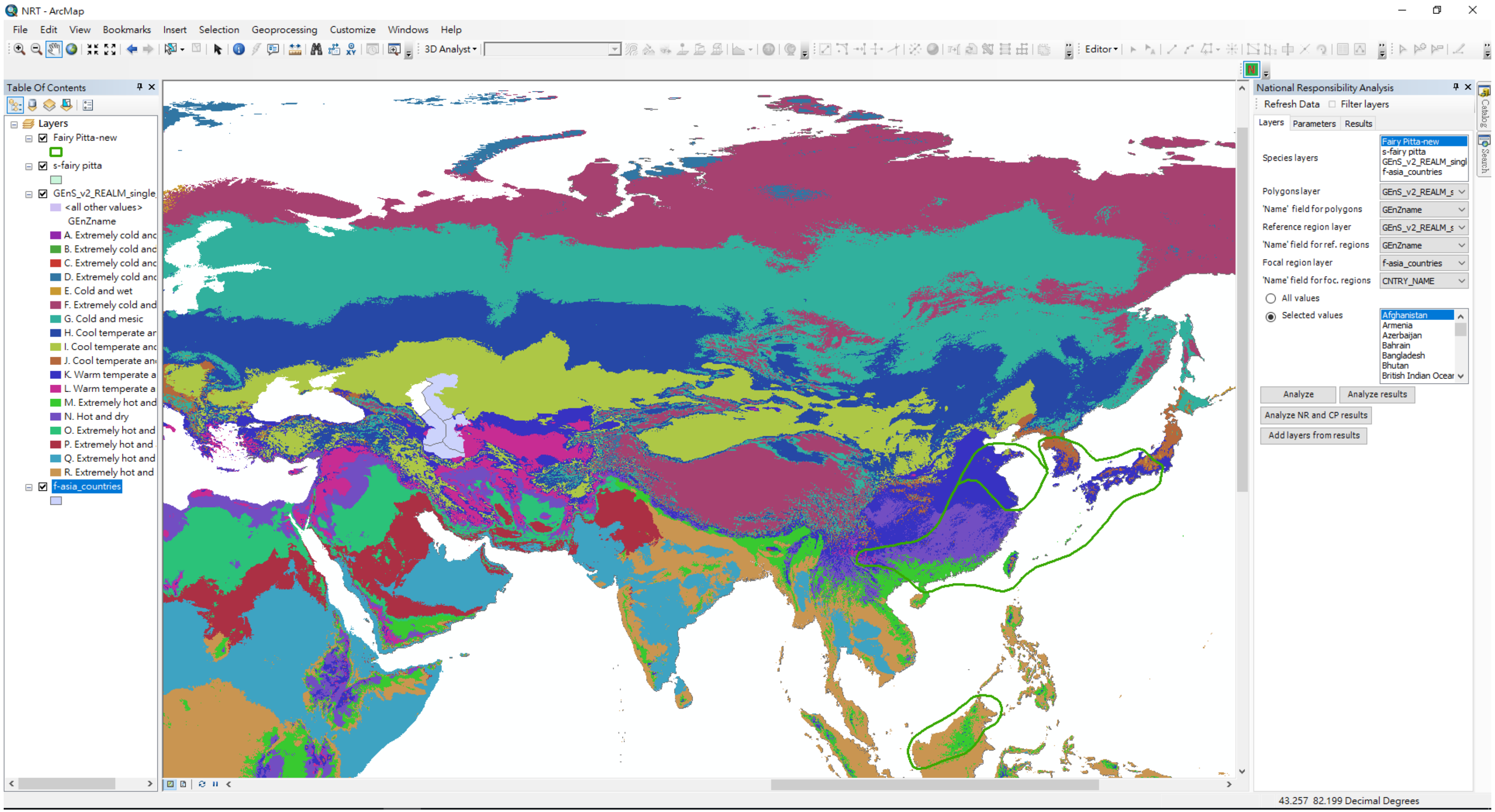

(a)

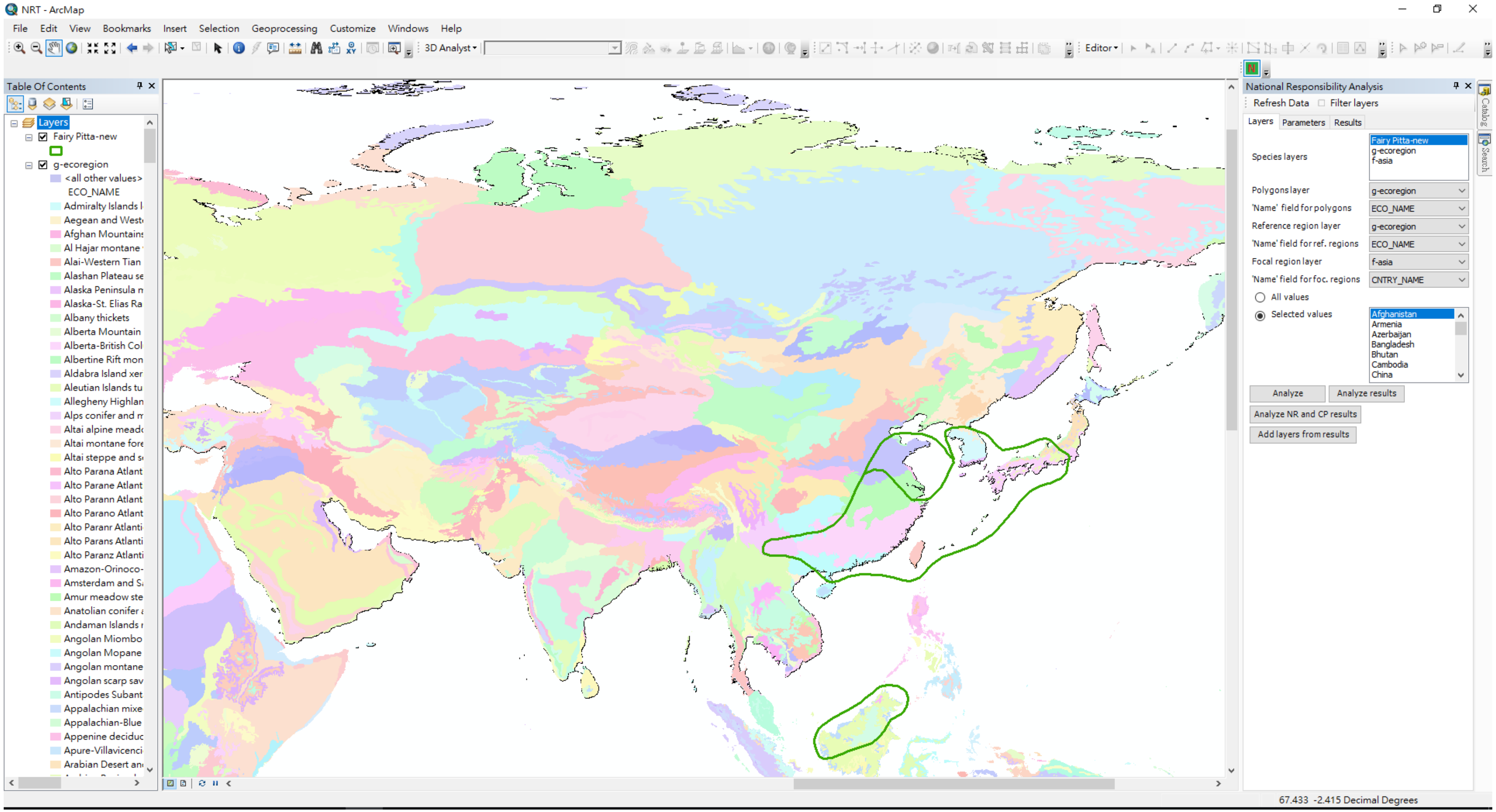
NRT - ArcMap
File Edit View Bookmarks Insert Selection Geoprocessing Customize Windows Help
3D Analyst
Editor
Table Of Contents
Layers
Fairy Pitta-new
g-ecoregion
<all other values>
ECO_NAME
Admiralty Islands l
Aegean and West
Afghan Mountains
Al Hajar montane
Alai-Western Tian
Alashan Plateau se
Alaska Peninsula m
Alaska-St. Elias Ra
Albany thickets
Alberta Mountain
Alberta-British Col
Albertine Rift mon
Aldabra Island xer
Aleutian Islands tu
Allegheny Highlan
Alps conifer and m
Altai alpine mead
Altai montane fore
Altai steppe and se
Alto Parana Atlant
Alto Parane Atlant
Alto Parann Atlant
Alto Parano Atlant
Alto Paranr Atlanti
Alto Parans Atlanti
Alto Paranz Atlanti
Amazon-Orinoco-
Amsterdam and S
Amur meadow ste
Anatolian conifer
Andaman Islands r
Angolan Miombo
Angolan Mopane
Angolan montane
Angolan scarp sav
Antipodes Subant
Appalachian mixe
Appalachian-Blue
Appenine deciduc
Apure-Villavicenci
Arabian Desert an
National Responsibility Analysis
Refresh Data
Filter layers
Layers
Parameters
Results
Species layers
Fairy Pitta-new
g-ecoregion
f-asia
Polygons layer
g-ecoregion
'Name' field for polygons
ECO_NAME
Reference region layer
g-ecoregion
'Name' field for ref. regions
ECO_NAME
Focal region layer
f-asia
'Name' field for foc. regions
CNTRY_NAME
All values
Selected values
Afghanistan
Armenia
Azerbaijan
Bangladesh
Bhutan
Cambodia
China
Analyze
Analyze results
Analyze NR and CP results
Add layers from results
Catalog
Search
67.433 -2.415 Decimal Degrees

(b)

Fig S1.2. Interface of the National Responsibility Tool in the ArcGIS platform

(a) Dialog to open input shapefiles for the selection of bio-regional layers (e.g. an GEnZ layer), reference layers, focal species layers (an example of which is shown in yellow on the maps), and focal area layers (right side), (b) dialog for setting the calculation parameter types used in the analysis of national responsibility (Polygon Count-Approach = PC-A or Polygon Area-Approach = PA-A), distribution parameters, a distribution factor, IUCN categories, and an output file (right side), and (c) display of a dialog box for checking the names of focal species.

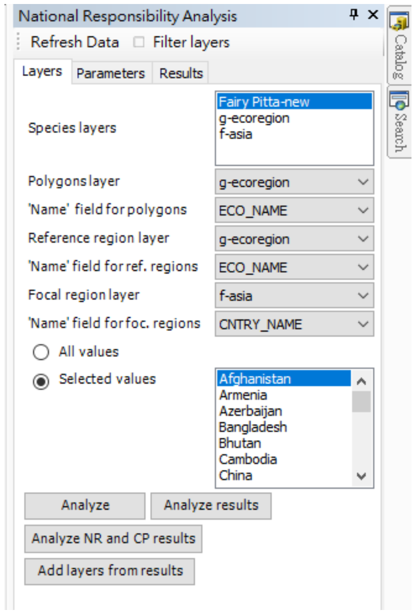

(a)

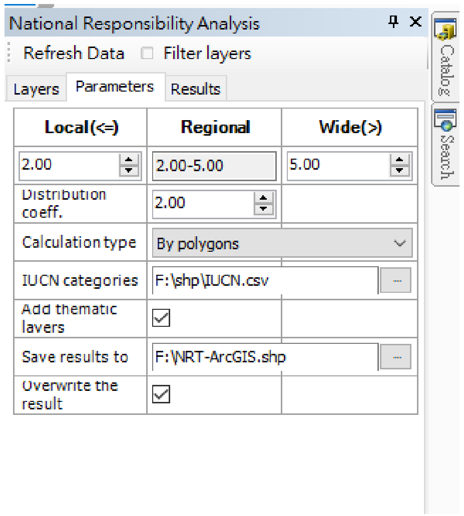

(b)

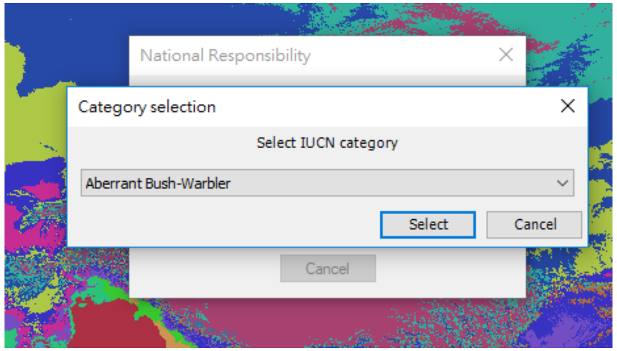

(c)

Fig S1.3. Interface of National Responsibility Tool in ArcGIS platform
(a) Display a tool processing bar, (b, c) display analysis maps with level and class legends (left side), and (d) browsing analysis results (right side).

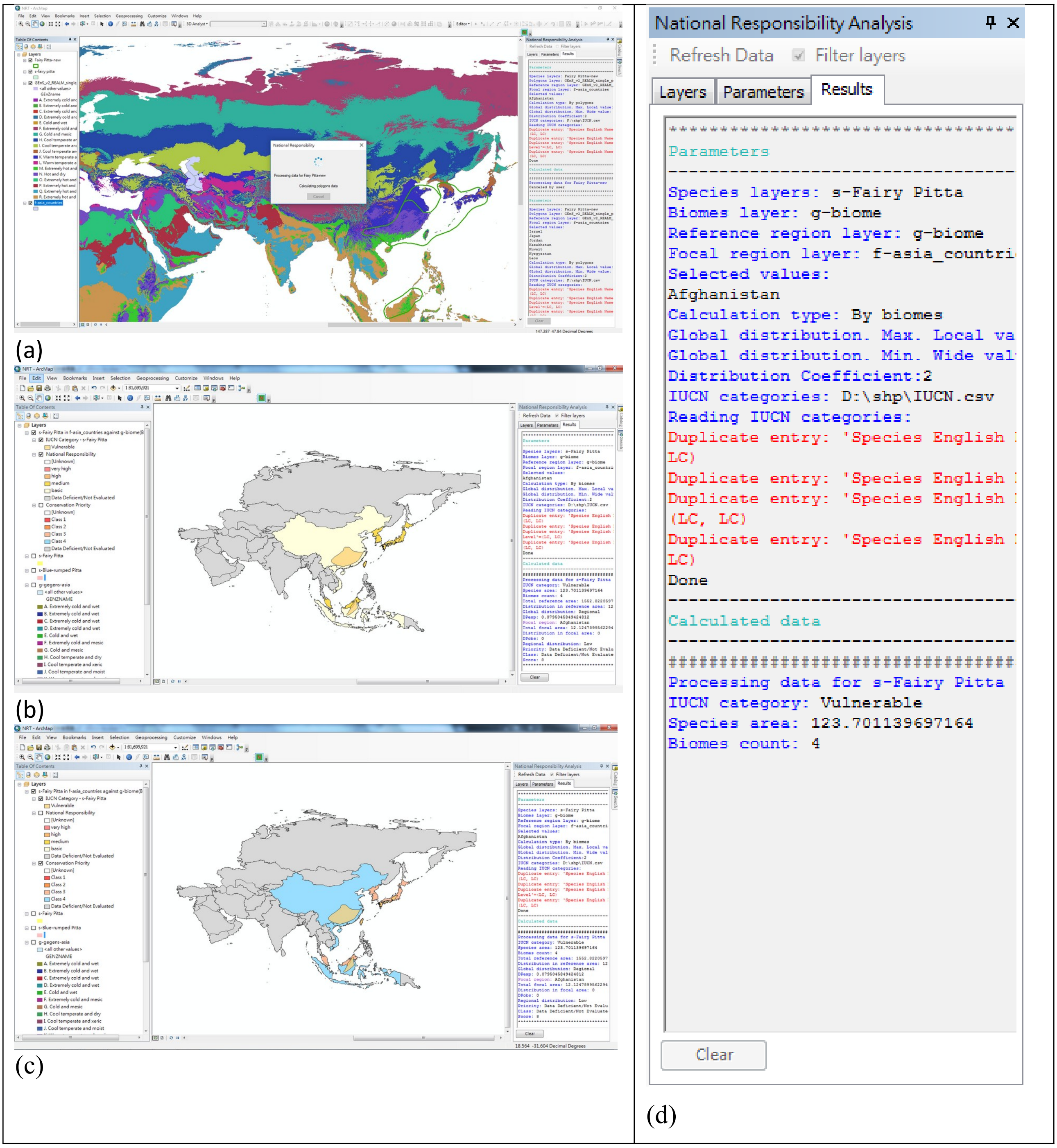

Fig S1.4. Interface of National Responsibility Tool in ArcGIS platform.

(a) A dialog box to create visual maps of national responsibility and conservation priority for multiple species, (b) example of a resulting map of national responsibility and conservation priority for multiple species (in this case 2), (c) a dialog box to create visual maps of national responsibility and conservation priority for all runs, (d) example of a map of the analyzed NR and CP results.

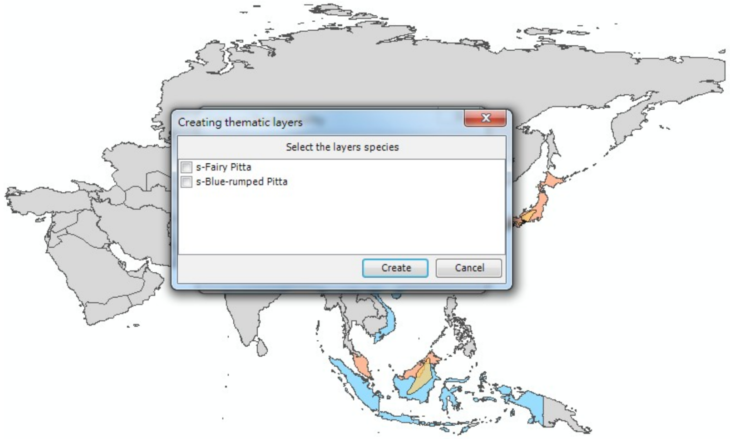

(a)

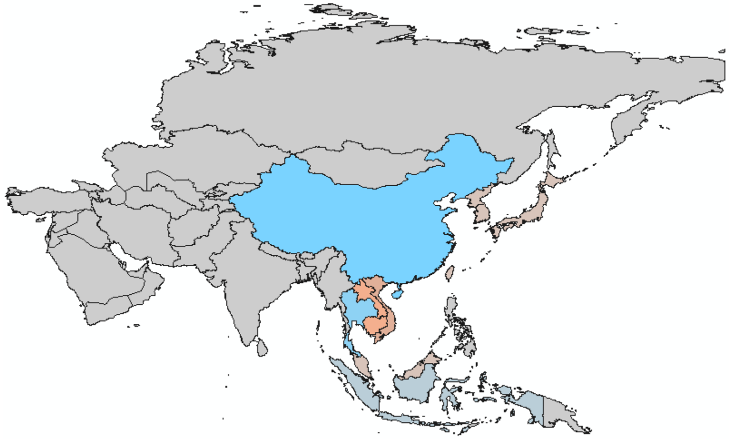

(b)

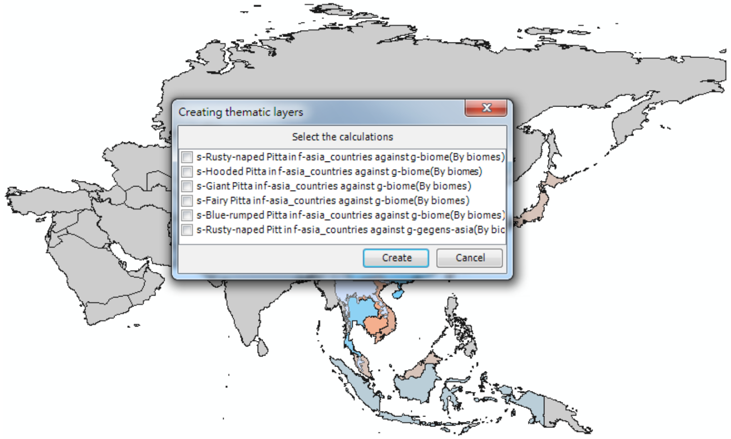

(c)

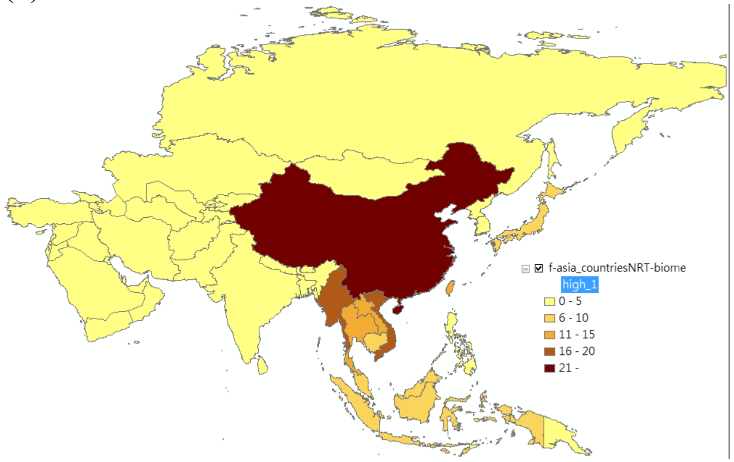

(d)

References

1. Metzger MJ, Bunce RG, Jongman RH, Sayre R, Trabucco A, Zomer R. A high resolution bioclimate map of the world: a unifying framework for global biodiversity research and monitoring. *Global Ecology and Biogeography* 2013;**22**(5):630-8.

2. Olson D, Dinerstein E, Wikramanayake E, Burgess N, Powell G, Underwood E, D'amico J, Itoua I, Strand H, Morrison J, Loucks C, Allnutt T, Ricketts T, Kura Y, Lamoreux J, Wettengel W, Hedao P, Kassem K. Terrestrial ecoregions of the world – A new map of life on Earth. *Bioscience* 2001;**51**(11):933-8.
