# Supplementary material for: A GIS-based policy support tool to determine national responsibilities and priorities for biodiversity conservation
Source: PLoS One. 2020 Dec 3;15(12):e0243135. doi: 10.1371/journal.pone.0243135 (PMC7714368; doi:10.1371/journal.pone.0243135)
Supplement: S2 File — (PDF) [file pone.0243135.s002.pdf]

Supporting Information S2 File: An example for the calculations of the Polygon Count-Approach and the Polygon Area-Approach

Fig S2.1 describes different types of relation between distribution areas and the number and area of biogeographic units (BUs) contained in the distribution areas. A species with a very small distribution area may only be found within a small BU (Fig S2.1a, E1). This is the case when the ecological niche of the species is strongly related to the environmental characteristics of the BU and both the extent of the BU and the spread of the species are limited by the same factors (e.g. on the peaks of isolated mountains). However, even if the range of a species is small, there may be overlaps with more than one BU (Fig S2.1a, E2). This is the case when environmental variability is high across the area, such as in mountainous areas with high altitude variability, but the boundaries of the species' niche are set by factors other than climatic or geographical conditions. Other examples are a large island with high environmental variability that is too isolated for the species under study to reach other islands or continents, or large valleys with dry and wet parts surrounded by a closed chain of high mountains. A species with a small distribution range may also occur in several BUs with different and partially large extents (Fig S2.1a, E3), e.g. if its dispersal is limited by other factors then those used for the definition of the BUs. Finally, a species with a small distribution range may be restricted to one very large BU (Fig S2.1a, E4). Examples are species that are restricted to a few islands within a large number of islands that occur in a given Biome.

An euryoecious species with a large distribution range may occur across many small BUs (Fig S2.1b, W1), e.g. mobile birds living in mountainous areas with high variability such as the Balkans or the Himalayas. Alternatively, the distribution may overlap with only a few BUs of larger extent (Fig S2.1b, W2). Finally, a large distribution area may be limited to only one large BU (Fig S2.1b, W3; e.g. if both the environmental characteristics and the ecological niche of the species are very closely related, but occur over a large spatial extent).

Fig S2.1. An example of the assessment of the pattern of relations between species area and the biogeographical units (BU) and of the classification for assessing the global distribution.

a) *The distribution of hypothetical species with a very small distribution area may be within only one small BU – E1 may overlap with several BUs of only small extent– E2, may overlap with several BUs of different and sometimes large extent – E3, or it may be located within a single large BU – E4.*

b) *The distribution of a species with a very large range may overlap with many BUs with different but rather small extent – W1, it may overlap with only a few BUs of larger extent – W2, or it may be within one single large BU – W3.*

c) *The distribution areas of the four hypothetical species E1-E4 are the same, but the area of the BUs enclosed in the distribution area ranges from very small to very large with a large jump in the percentage between the species E1, E2 and E3, E4, while the number of overlapped BUs (for E1=1, E2=3, E3=3, and E4=1), does not correspond to the size of the BU areas overlapped.*

d) *The ranges of distribution of the three widespread hypothetical species W1-W3 are the same, but the areas of the overlapped BUs ranges from very small to very large with a large jump in the dimensions between the species W1, W2 and W3, while the number of overlapped BUs (for W1=8, W2=3, W3=1) is not related to the size of the BU areas.*

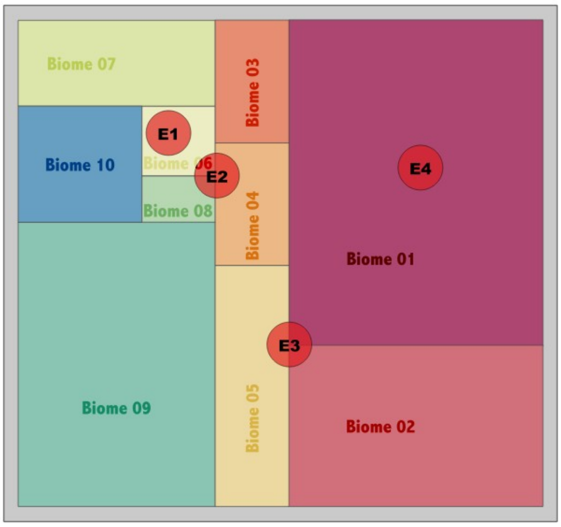

a)

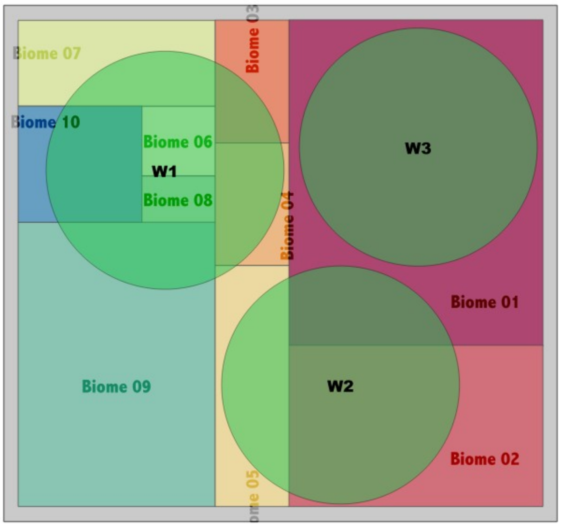

b)

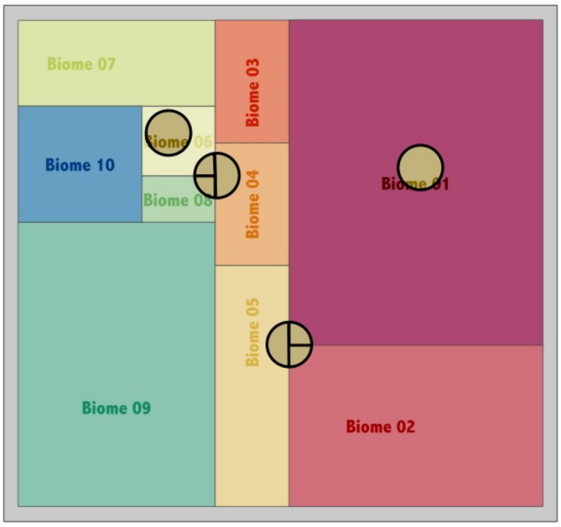

c)

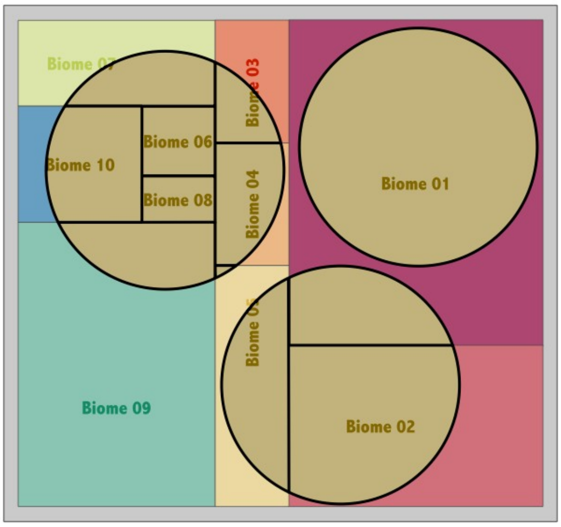

d)

In most of these cases, the number of BUs that overlap with the species' distribution areas is not closely related to the sum of the areas of the respective BUs. Table S2.1 shows the counts and areas resulting from the pattern of relations shown in Fig 2.1.

Table S2.1. Pattern of relationships between the distribution area of the hypothetical species of Fig S2.1 (E1, E2, E3, E4, W1, W2, W3) and the Biogeographic Units (BU). *Proportion: Biome area divided by total area.*

**Biogeographic Unit**	**Area in km²**	**Proportion**	**E1**	**E2**	**E3**	**E4**	**W1**	**W2**	**W3**
**Biome 01**	3,237.71	32.38%			1	1		1	1
**Biome 02**	1,605.76	16.06%			1			1	
**Biome 03**	357.17	3.57%					1		
**Biome 04**	354.68	3.55%		1			1		
**Biome 05**	697.68	6.98%			1		1	1	
**Biome 06**	202.07	2.02%	1	1			1		
**Biome 07**	659.93	6.60%					1		
**Biome 08**	135.42	1.35%		1			1		
**Biome 09**	2,187.46	21.87%					1		
**Biome 10**	562.13	5.62%					1		
**Number of BUs included in the range**			1	3	3	1	8	3	1
**Sum Area**	10000		202.07	692.17	5,541.14	3,237.71	5,156.53	5,541.14	3,237.71
**Sum Proportions**		100.00%	2.02%	6.92%	55.41%	32.38%	51.57%	55.41%	32.38%

It needs more research to find out which way is the most appropriate for the assessment of the global distribution. This is the reason why we developed and implemented the Polygon Area-Approach into the ArcGIS-NRA-tool.
