# Supplementary material for: A GIS-based policy support tool to determine national responsibilities and priorities for biodiversity conservation
Source: PLoS One. 2020 Dec 3;15(12):e0243135. doi: 10.1371/journal.pone.0243135 (PMC7714368; doi:10.1371/journal.pone.0243135)
Supplement: S3 File — (ZIP) [file pone.0243135.s003.zip › S3_File_List_of_species.pdf]

Supporting Information S3: List of species used in the illustration example

Table S3.1: List of common names of bird species used in the analysis.

```
Species_Scientific_Name;Species_English_Name;IUCN_Level
Pyrrhoplectes epauletta;Gold-naped Finch;LC
Pyrrhula erythaca;Gray-headed Bullfinch;LC
Coccothraustes coccothraustes;Hawfinch;LC
Mycerobas affinis;Collared Grosbeak;LC
Pyrrhula pyrrhula;Eurasian Bullfinch;LC
Carduelis ambigua;Black-headed Greenfinch;LC
Pyrrhula nipalensis;Brown Bullfinch;LC
Carduelis cannabina;Eurasian Linnet;LC
Carduelis spinus;Eurasian Siskin;LC
Eophona personata;Japanese Grosbeak;LC
Carduelis chloris;European Greenfinch;LC
Carpodacus rubicilla;Great Rosefinch;LC
Carpodacus pulcherrimus;Beautiful Rosefinch;LC
Carpodacus nipalensis;Dark-breasted Rosefinch;LC
Serinus pusillus;Fire-fronted Serin;LC
Leucosticte arctoa;Asian Rosy-Finch;LC
Leucosticte brandti;Black-headed Mountain-Finch;LC
Fringilla montifringilla;Brambling;LC
Fringilla coelebs;Chaffinch;LC
Carpodacus edwardsii;Dark-rumped Rosefinch;LC
Montifringilla adamsi;Black-winged Snowfinch;LC
Gracula religiosa;Common Hill Myna;LC
Ampeliceps coronatus;Golden-crested Myna;LC
Ploceus philippinus;Baya Weaver;LC
Ploceus benghalensis;Bengal Weaver;LC
Aplonis panayensis;Asian Glossy Starling;LC
Corvus corone;Carrion Crow;LC
Corvus torquatus;Collared Crow;NT
Corvus dauuricus;Daurian Jackdaw;LC
Acridotheres fuscus;Jungle Myna;LC
Corvus splendens;House Crow;LC
Corvus macrorhynchos;Large-billed Crow;LC
Pica pica;Eurasian Magpie;LC
Nucifraga caryocatactes;Eurasian Nutcracker;LC
Corvus monedula;Eurasian Jackdaw;LC
Urocissa erythrorhyncha;Blue Magpie;LC
```

Urocissa caerulea;Formosan Magpie;LC
Urocissa flavirostris;Gold-billed Magpie;LC
Dendrocitta formosae;Gray Treepie;LC
Artamus fuscus;Ashy Woodswallow;LC
Dicrurus macrocercus;Black Drongo;LC
Dicrurus aeneus;Bronzed Drongo;LC
Lanius sphenocercus;Chinese Gray Shrike;LC
Cissa chinensis;Green Magpie;LC
Dicrurus leucophaeus;Ashy Drongo;LC
Lanius tephronotus;Gray-backed Shrike;LC
Dicrurus paradiseus;Greater Racket-tailed Drongo;LC
Dicrurus hottentottus;Hair-crested Drongo;LC
Tephrodornis gularis;Large Woodshrike;LC
Dicrurus annectans;Crow-billed Drongo;LC
Oriolus xanthornus;Black-hooded Oriole;LC
Lanius cristatus;Brown Shrike;LC
Lanius bucephalus;Bull-headed Shrike;LC
Lanius collurioides;Burmese Shrike;LC
Oriolus chinensis;Black-naped Oriole;LC
Oriolus oriolus;Eurasian Golden Oriole;LC
Dicaeum ignipectus;Fire-breasted Flowerpecker;LC
Lanius minor;Lesser Gray Shrike;LC
Dicrurus remifer;Lesser Racket-tailed Drongo;LC
Irena puella;Asian Fairy-bluebird;LC
Certhia familiaris;Eurasian Treecreeper;LC
Sitta magna;Giant Nuthatch;VU
Sitta nagaensis;Chestnut-vented Nuthatch;LC
Sitta europaea;Eurasian Nuthatch;LC
Pseudopodoces humilis;Ground Tit;LC
Parus xanthogenys;Black-lored Tit;LC
Parus major;Great Tit;LC
Zosterops japonicus;Japanese White-eye;LC
Aethopyga saturata;Black-throated Sunbird;LC
Aethopyga siparaja;Eastern Crimson Sunbird;LC
Aethopyga ignicauda;Fire-tailed Sunbird;LC
Aethopyga christinae;Fork-tailed Sunbird;LC
Aethopyga nipalensis;Green-tailed Sunbird;LC
Arachnothera longirostra;Little Spiderhunter;LC
Certhia discolor;Brown-throated Treecreeper;LC

Remiz consobrinus;Chinese Penduline-Tit;LC
Remiz pendulinus;Eurasian Penduline-Tit;LC
Cephalopyrus flammiceps;Fire-capped Tit;LC
Certhia himalayana;Bar-tailed Treecreeper;LC
Sitta formosa;Beautiful Nuthatch;VU
Aegithalos concinnus;Black-throated Tit;LC
Paradoxornis alphonsianus;Ashy-throated Parrotbill;LC
Paradoxornis flavirostris;Black-breasted Parrotbill;VU
Paradoxornis nipalensis;Black-throated Parrotbill;LC
Paradoxornis unicolor;Brown Parrotbill;LC
Paradoxornis brunneus;Brown-winged Parrotbill;LC
Paradoxornis gularis;Gray-headed Parrotbill;LC
Parus monticolus;Green-backed Tit;LC
Paradoxornis atrosuperciliaris;Black-browed Parrotbill;LC
Aegithalos iouschistos;Black-browed Tit;LC
Myzornis pyrrhoura;Fire-tailed Myzornis;LC
Heterophasia pulchella;Beautiful Sibia;LC
Heterophasia melanoleuca;Black-backed Sibia;LC
Heterophasia desgodinsi;Black-headed Sibia;LC
Alcippe brunneicauda;Brown Fulvetta;NT
Alcippe poioicephala;Brown-cheeked Fulvetta;LC
Paradoxornis zappeyi;Gray-hooded Parrotbill;VU
Conostoma oemodium;Great Parrotbill;LC
Panurus biarmicus;Bearded Reedling;LC
Yuhina nigrimenta;Black-chinned Yuhina;LC
Minla strigula;Chestnut-tailed Minla;LC
Alcippe striaticollis;Chinese Fulvetta;LC
Alcippe variegaticeps;Gold-fronted Fulvetta;VU
Actinodura nipalensis;Hoary-throated Barwing;LC
Pteruthius rufiventer;Black-headed Shrike-Babbler;LC
Pteruthius aenobarbus;Chestnut-fronted Shrike-Babbler;LC
Babax lanceolatus;Chinese Babax;LC
Alcippe brunnea;Dusky Fulvetta;LC
Heterophasia gracilis;Gray Sibia;LC
Alcippe morrisonia;Gray-cheeked Fulvetta;LC
Spelaeornis troglodytoides;Bar-winged Wren-Babbler;LC
Stachyris pyrrhops;Black-chinned Babbler;LC
Stachyris ambigua;Buff-chested Babbler;LC
Timalia pileata;Chestnut-capped Babbler;LC

Stachyris chrysaea;Golden Babbler;LC
Stachyris nigriceps;Gray-throated Babbler;LC
Pomatorhinus ferruginosus;Coral-billed Scimitar-Babbler;LC
Cutia nipalensis;Cutia;LC
Babax waddelli;Giant Babax;NT
Pteruthius xanthochlorus;Green Shrike-Babbler;LC
Garrulax affinis;Black-faced Laughingthrush;LC
Garrulax squamatus;Blue-winged Laughingthrush;LC
Garrulax erythrocephalus;Chestnut-crowned Laughingthrush;LC
Garrulax canorus;Hwamei;LC
Garrulax lunulatus;Barred Laughingthrush;LC
Napothera epilepidota;Eyebrowed Wren-Babbler;LC
Pnoepyga immaculata;Immaculate Wren-Babbler;LC
Rimator malacoptilus;Long-billed Wren-Babbler;LC
Liocichla omeiensis;Gray-faced Liocichla;VU
Pomatorhinus hypoleucos;Large Scimitar-Babbler;LC
Hypothymis azurea;Black-naped Monarch;LC
Garrulax maesi;Gray Laughingthrush;LC
Garrulax pectoralis;Greater Necklaced Laughingthrush;LC
Terpsiphone atrocaudata;Japanese Paradise-Flycatcher;NT
Garrulax monileger;Lesser Necklaced Laughingthrush;LC
Oenanthe deserti;Desert Wheatear;LC
Cochoa viridis;Green Cochoa;LC
Garrulax chinensis;Black-throated Laughingthrush;LC
Garrulax maximus;Giant Laughingthrush;LC
Terpsiphone paradisi;Asian Paradise-Flycatcher;LC
Cinclidium frontale;Blue-fronted Robin;LC
Grandala coelicolor;Grandala;LC
Enicurus scouleri;Little Forktail;LC
Phoenicurus alaschanicus;Ala Shan Redstart;NT
Phoenicurus ochruros;Black Redstart;LC
Tarsiger johnstoniae;Collared Bush-Robin;LC
Phoenicurus auroreus;Daurian Redstart;LC
Oenanthe isabellina;Isabelline Wheatear;LC
Enicurus immaculatus;Black-backed Forktail;LC
Phoenicurus frontalis;Blue-fronted Redstart;LC
Culicicapa ceylonensis;Gray-headed Canary-Flycatcher;LC
Luscinia brunnea;Indian Blue Robin;LC
Erithacus akahige;Japanese Robin;LC

Cyornis rubeculoides;Blue-throated Flycatcher;LC
Niltava davidi;Fujian Niltava;LC
Cyornis hainanus;Hainan Blue-Flycatcher;LC
Tarsiger chrysaeus;Golden Bush-Robin;LC
Luscinia obscura;Black-throated Blue Robin;VU
Luscinia megarhynchos;Common Nightingale;LC
Luscinia pectardens;Firethroat;NT
Muscicapa ferruginea;Ferruginous Flycatcher;LC
Ficedula zanthopygia;Korean Flycatcher;LC
Ficedula westermanni;Little Pied Flycatcher;LC
Muscicapa dauurica;Asian Brown Flycatcher;LC
Sylvia nana;Asian Desert Warbler;LC
Cyornis banyumas;Hill Blue-Flycatcher;LC
Niltava grandis;Large Niltava;LC
Cyanoptila cyanomelana;Blue-and-white Flycatcher;LC
Sylvia nisoria;Barred Warbler;LC
Muscicapa muttui;Brown-breasted Flycatcher;LC
Sylvia curruca;Lesser Whitethroat;LC
Abroscopus schisticeps;Black-faced Warbler;LC
Tickellia hodgsoni;Broad-billed Warbler;LC
Seicercus castaniceps;Chestnut-crowned Warbler;LC
Seicercus poliogenys;Gray-cheeked Warbler;LC
Phylloscopus coronatus;Eastern Crowned-Warbler;LC
Phylloscopus emeiensis;Emei Leaf-Warbler;LC
Muscicapa sibirica;Dark-sided Flycatcher;LC
Muscicapa griseisticta;Gray-streaked Flycatcher;LC
Sylvia communis;Greater Whitethroat;LC
Phylloscopus maculipennis;Ashy-throated Warbler;LC
Phylloscopus pulcher;Buff-barred Warbler;LC
Phylloscopus yunnanensis;Chinese Leaf-Warbler;LC
Phylloscopus kansuensis;Gansu Leaf-Warbler;LC
Phylloscopus trochiloides;Greenish Warbler;LC
Phylloscopus proregulus;Lemon-rumped Warbler;LC
Seicercus burkii;Golden-spectacled Warbler;LC
Seicercus tephrocephalus;Gray-crowned Warbler;LC
Phylloscopus hainanus;Hainan Leaf-Warbler;VU
Phylloscopus magnirostris;Large-billed Leaf-Warbler;LC
Acrocephalus bistrigiceps;Black-browed Reed-Warbler;LC
Acrocephalus concinens;Blunt-winged Warbler;LC

Hippolais caligata;Booted Warbler;LC
Acrocephalus stentoreus;Clamorous Reed-Warbler;LC
Hippolais pallida;Eastern Olivaceous Warbler;LC
Acrocephalus scirpaceus;Eurasian Reed-Warbler;LC
Phylloscopus subaffinis;Buff-throated Warbler;LC
Orthotomus sutorius;Common Tailorbird;LC
Leptopoecile elegans;Crested Tit-Warbler;LC
Orthotomus atrogularis;Dark-necked Tailorbird;LC
Bradypterus major;Long-billed Bush-Warbler;NT
Cettia flavolivacea;Aberrant Bush-Warbler;LC
Urosphena squameiceps;Asian Stubtail;LC
Cettia fortipes;Brownish-flanked Bush-Warbler;LC
Cettia major;Chestnut-crowned Bush-Warbler;LC
Tesia castaneocoronata;Chestnut-headed Tesia;LC
Tesia cyaniventer;Gray-bellied Tesia;LC
Bradypterus luteoventris;Brown Bush-Warbler;LC
Locustella naevia;Grasshopper Warbler;LC
Locustella lanceolata;Lanceolated Warbler;LC
Prinia atrogularis;Hill Prinia;LC
Brachypteryx leucophrys;Lesser Shortwing;LC
Turdus chrysolaus;Brown-headed Thrush;LC
Prinia hodgsonii;Gray-breasted Prinia;LC
Cettia brunnifrons;Gray-sided Bush-Warbler;LC
Prinia polychroa;Brown Prinia;LC
Turdus mupinensis;Chinese Thrush;LC
Turdus naumanni;Dusky Thrush;LC
Turdus feae;Gray-sided Thrush;VU
Turdus poliocephalus;Island Thrush;LC
Turdus cardis;Japanese Thrush;LC
Monticola solitarius;Blue Rock-Thrush;LC
Myophonus caeruleus;Blue Whistling-Thrush;LC
Turdus rubrocanus;Chestnut Thrush;LC
Turdus ruficollis;Dark-throated Thrush;LC
Turdus merula;Eurasian Blackbird;LC
Turdus obscurus;Eyebrowed Thrush;LC
Turdus hortulorum;Gray-backed Thrush;LC
Zoothera monticola;Long-billed Thrush;LC
Prunella collaris;Alpine Accentor;LC
Prunella atrogularis;Black-throated Accentor;LC

Prunella fulvescens;Brown Accentor;LC
Aegithina lafresnayei;Great Iora;LC
Prunella himalayana;Himalayan Accentor;LC
Monticola cinclorhynchus;Blue-capped Rock-Thrush;LC
Monticola rufiventris;Chestnut-bellied Rock-Thrush;LC
Zoothera marginata;Dark-sided Thrush;LC
Myophonus insularis;Formosan Whistling-Thrush;LC
Hemixos castanonotus;Chestnut Bulbul;LC
Aegithina tiphia;Common Iora;LC
Regulus goodfellowi;Flamecrest;LC
Regulus regulus;Goldcrest;LC
Chloropsis aurifrons;Golden-fronted Leafbird;LC
Iole propinqua;Gray-eyed Bulbul;LC
Bombycilla japonica;Japanese Waxwing;NT
Hypsipetes leucocephalus;Black Bulbul;LC
Chloropsis cochinchinensis;Blue-winged Leafbird;LC
Ixos amaurotis;Brown-eared Bulbul;LC
Pericrocotus divaricatus;Ashy Minivet;LC
Hemipus picatus;Bar-winged Flycatcher-shrike;LC
Pericrocotus cantonensis;Brown-rumped Minivet;LC
Spizixos semitorques;Collared Finchbill;LC
Spizixos canifrons;Crested Finchbill;LC
Pycnonotus melanicterus;Black-crested Bulbul;LC
Pycnonotus atriceps;Black-headed Bulbul;LC
Pycnonotus xanthorrhous;Brown-breasted Bulbul;LC
Pycnonotus flavescens;Flavescent Bulbul;LC
Pycnonotus sinensis;Light-vented Bulbul;LC
