# Supplementary material for: A GIS-based policy support tool to determine national responsibilities and priorities for biodiversity conservation
Source: PLoS One. 2020 Dec 3;15(12):e0243135. doi: 10.1371/journal.pone.0243135 (PMC7714368; doi:10.1371/journal.pone.0243135)
Supplement: S4 File — (PDF) [file pone.0243135.s004.pdf]

Supporting Information S4 File: Performance and possibilities for optimization

The GEnZ shapefile [1] contains 125 strata with a spatial resolution of approximately 1 km^2 and can be downloaded for non-commercial purposes from [2]. Principle Component Analysis (PCA) was used in the classifications of Metzger et al. [1] to construct the environmental stratification across the world using on 24 environmental variables (see detail in [1,3]). Some of the polygons within the GEnZ shapefile may have a very irregular shape with many small islands and gaps within larger polygons. Technically speaking, these objects can be stored either as so-called multi-part polygons (i.e., one object consisting of at least two or more small objects stored under only one ID and with only one row in the attribute table) or as combined single-part polygons (each small separate unit is a separate object with its unique ID and a corresponding row in the attribute table, with additional data on area, perimeter, etc.). In our experience, single-part polygons perform better in analyses than multi-part polygons, but multi-part polygons provide results that are easier to interpret. This is especially true for the FA. Some countries, such as Russia, Greece or Indonesia, have a many small islands. In single-part mode, the results are calculated separately for each of the small islands, while in multi-part mode, a single result is generated for the whole country, considerably reduces the computation time.

In addition, features of the global environmental stratification map consist of some areas with many artificial stair-shaped borders, which lead to very time-consuming operations. Therefore, we tried to reduce the accuracy and generalize the boundaries to considerably shorten the computation time. However, such a generalization is a compromise between accuracy and computation time. For example, maps with generalized boundaries of small regions with very irregular shapes, such as areas in the Himalayas or other regions with very high climatic diversity, do not take into account the entire environmental variability. For analyses that focus on species living in such regions, we strongly recommend using the original files although this will increase the computation time.

References

1. Metzger MJ, Bunce RG, Jongman RH, Sayre R, Trabucco A, Zomer R. A high resolution bioclimate map of the world: a unifying framework for global biodiversity research and monitoring. *Global Ecology and Biogeography* 2013;**22**(5):630-8.

2. Metzger MJ. The Global Environmental Stratification: A high-resolution bioclimate map of the world [dataset]. The University of Edinburgh; 2018. https://doi.org/10.7488/ds/2354.

3. Metzger MJ, Bunce RGH, Jongman RHG, Mucher CA, Watkins JW. A climatic stratification of the environment of Europe. *Global Ecology and Biogeography* 2005;**14**:549–63.
