# Supplementary material for: A GIS-based policy support tool to determine national responsibilities and priorities for biodiversity conservation
Source: PLoS One. 2020 Dec 3;15(12):e0243135. doi: 10.1371/journal.pone.0243135 (PMC7714368; doi:10.1371/journal.pone.0243135)
Supplement: Attachment — Submitted filename: 20200921-Response2Reviewers.pdf [file pone.0243135.s008.pdf]

Summary of revisions and amendments and detailed response to comments and questions of the reviewers

We have overhauled most of the sections in our text based on your and the reviewers comments, provided more detailed information about the conceptual framework on which the ArcGIS NRA-tool is based, reduced the number of acronyms used, explained the basics of this framework in order to make clear how this tool works, and we tried to adapt the description of the software to those readers that are not familiar with this framework. You will find a detailed response to each point raised by you and the reviewers below these lines.

We would like to emphasize that it is our main aim to present a software tool for research. The research results provided with our manuscript are rather meant as an example and an illustration of the utility and usability of this tool but not as a sophisticated analysis of the national responsibilities countries do have for threatened bird species in Asia and where the main priorities for conservation effort should be set.

We tried to make sure that the manuscript meets PLOS ONE criteria for manuscripts that describe new software for applications following the guidelines mentioned in your decision letter from April 21st of this year.

Utility: From our knowledge, there is no other GIS tool available, which is based on the methods presented by Schmeller et al. in several publications mentioned as references in our manuscript. After publication in PLOS ONE we will open opportunities for active collaboration and further development and overhand the complete source code to the public via github and/or sourceforge securing long term availability and maintenance. The future maintenance, software development and growth will depend on the interest of the scientific community, therefore.

Validation: Until now, there was no option for the automatization and detailed documentation of analyses of National Responsibilities and Conservation Priorities based on the methods described by Schmeller et al. With our ArcGIS-NRA-Tool, these partly difficult and especially time consuming steps of the analyses can be done automatically as demonstrated with the examples described in our manuscript.

Availability: The tool is currently hosted in https://github.com/ and will be made publicly available via https://sourceforge.net/ under the GNU General Public Licence after our manuscript is accepted. The links will be updated in the final version of the manuscript, permissions will be set to "public", and the source code will be made available. Besides, our tool is already advertised in the web presentation of the EH FP7 SCALES project (www.scales-project.net) and at the web presentation of the SCALES-Taiwan project (http://homepage.ntu.edu.tw/~yplin/Scales-Taiwan.htm). We will amend the links in these web pages too. A long term backup will be made in the archive of the Helmholtz Centre for Environmental Research (UFZ) in Leipzig.

Reviewer #1:

This manuscript regards the application of a prioritization method together with the national responsibility approach (an approach already used in several scientific works) in a novel GIS tool. This tool is presented with data from Asia. The manuscript is well written but there are a number of major concerns that need to be addressed.

I think that national responsibility and conservation priority approaches should be thoroughly presented. What is national responsibility? How can this be derived? This in addition to the specific explanation given in the method section. There are a number of prioritization methods (including or not responsibility) applied in the literature and a brief overview should be given (see also indications in the discussion section). Data availability and type can influence the applicability of spatial prioritization approaches. This should be introduced as it can be relevant for application of the tool to other regions.

Thank you for the suggestions. We have changed this part in the introduction and extended it by more detailed explanations about national responsibility and approaches for the determination of conservation priorities. However, it is not the aim of our paper to review such approaches but to present a software tool that can be used to perform a certain type of analyses based on methods already described before. This tool should not be used without respective knowledge and experience in this field as it is required for any scientific work.

Methods

There are some aspects that deserve further explanation. Please give more details on what is meant with focal and reference area (I don't think referring to Schmeller and colleagues suffices). Regarding the additional factor in the

prioritization process, I miss how the different scores and classes were decided and assigned. I think that a graphical framework of the National Responsibility Assessment Tool would enable a better understanding of the type of data, steps and requirements needed for its application (maybe integrated in fig. 1).

We have overhauled the whole section and provided new figures and maps explaining the different terms and the process. There is already a graphical framework explaining the workflow (Fig. 2) and the user interface as well as the data needed (S2). We think that a potential user should study all this material provided by us in detail and read the respective references to get familiar with the approaches and the program first. Again, it is not the aim of this paper to explain and discuss approaches for the determination of national responsibilities, we want to introduce a tool. We also separated the more technical parts from the main text and provided them in the supplemental material to make the paper easier to read.

L119-120: I suggest anticipating that this can be used at the global as well as at other scales (regional? national?).

We stated this in an introductory sentence referring to the special case of global analyses and the data needed for this. At the end of the paragraph we were writing:

129 However, analyses can be conducted also at other scales and with other administrative
130 references.

We moved this sentence to the beginning and inserted a link to Supplement S1 to make this clearer.

L126-127: I do not understand what files authors refer to. The message of this sentence is not clear.

The approach requires the definition of the Reference Area. The borders of this area can be set either by a special file (e.g. created by a dissolve operation based on a shapefile of the continents of the world) or just by referring in the respective fields in the user interface to either the file used for the Geographical Regions or the Ecological Unit/Region. We suggest to have a glance on Supplement S1 while reading this section because this document shows the respective screenshots of the user interface. We refer to S1 in this paragraph now.

L127: "All layers resp. GIS" is not clear.

We agree that this part of the sentence may be confusing and have tried to write more clearly what we mean. Thank you!

I found really complex to follow the "Focal area and reference area" section. A table could help in reporting what are the different options.

We now explain this part more in detail with a more detailed description of the method and a set of examples in five new figures.

One additional suggestion is to add the factors used in the applied example(see comment on S1). This would enable also to use as example specific species. It is not clear whether species data can be points, polygons and/or based on grids.

Thank you. However, we clearly wrote:

119 The ArcGIS-NRA-Tool needs four predefined layers with polygons to allow assessments …

It is not possible to use data organized in points or grids, this is not a limitation. It would be just inappropriate to use such type of data with this kind of geographical method.

Even though I do not have a technical background regarding ArcGIS, there is one important point from my side. This tool works in ArcGIS that is a private platform. There are many other programmes such as QGIS and R statistics that are open source and can be accessed without any restrictions. Why didn't authors decided t produce tools or scripts for open source programmes? I think this would help the application of the approach.

Thank you. We agree with this opinion. For R we don't see any necessity to provide a special program because an experienced user can do this very easily by itself. ArcGIS is widely used in administrations and scientific institutions and also by organisations like e.g., the IUCN. Therefore we started the development of such a tool with ArcGIS. At this time QGIS was not that widespread and commonly used as it is today. However, we also have already prepared a version for QGIS, which is still in a testing phase but will be released soon too. In the section "Further development" we have added a respective paragraph to the manuscript.

L259-260: It is not clear if one single biogeographic category is assigned to each country.

As mentioned already before, we have tried to explain this part more in detail with a more detailed description of the method and a set of examples in five new figures. The tool provides two different approaches, one is using simple counting of the number of polygons, which are overlapped / touched by the species area and a certain country the other one is performing an intersection among the different layers to calculate the accurate proportions of the overlapping areas.

L260-261: Which scientific reference is used to identify widespread and migratory species?

We didn't use any reference for that. This sentences simply mean that we didn't exclude species that are either living in areas bigger than the chosen Reference Area (Asia) = widespread and/or are migratory species, means leaving the Reference Area at least for parts of the year. However, it was not the aim of this study to discuss special aspects of national responsibility assessments related to migration or animal movement, we only want to give an example for the use of our tool. We added a short sentence about this to the manuscript.

Fig. 3: I do not think this map is relevant to understand the proposed process or to show its results! This could be part of one of the Supplementary files.

Thank you for the suggestion. We agree and have dropped this figure from the manuscript.

Overall I think that acronyms do not help the reader.

Thank you! We agree. We only used the abbreviations to keep the text shorter. Because PLOS ONE has no word limits we decided to use abbreviations only if we think it is helpful.

L273: Are the ranges of the number of biogeographic zones used to differentiate between local, regional or widely distributed species fixed? If these are fixed, as I have understood, such ranges should be reported in the method section and removed from the results.

Thank you! We agree and have removed this information from the results but referred to references and a figure in the methods section.

Fig. 4: Not clear what the numbers reported in the figure legend mean. The caption misses this information and what the different colours mean.

Thank you! We have revised and improved this Figure (now Figure 8)

Fig. 5: Not clear what the numbers reported in the figure legend mean. The caption misses this information and what the different colours mean.

Thank you! We have revised and improved this Figure (now Figure 9)

L308-309: Friendly and attractive environment! I think authors can state this once they have users' feedback.

Thank you! We agree and have deleted this statement from the text.

Results for the study case show that in some cases there can be a strong dependency on the national responsibility category (i.e. on species distribution) as many species usually are classified as least concerned. A discussion of this and other possible limitations should be integrated in the discussion.

We don't understand this sentence. The methodological approach and possible limitations were already addressed in the methodological papers. This is not the aim of our manuscript. There are no such limitations related directly to the tool. Additionally, we have discussed special aspects in Lines 312 to 322 in the revised manuscript.

L323-328: It would be nice to have some results on this overlap in the supplementary material.

Thank you for the suggestion. This might be a misunderstanding. We didn't do any special analyses in this regard. We only linked our results to information provided by the papers cited. We revised this paragraph to make this more clear. However, now we provide still more data from the output files produced by our tool (Supplement S5) and some statistics, which will be presented in an additional table in the manuscript (Table 2), and supported by Supplement S6. This material is used for a more detailed interpretation, which we have added to this section.

I suggest to highlight the possibility or not of adding some indicators of conservation priority. For example, current and future acting forces can help to set priorities also in terms of conservation and management activities. There are a number of works that use these factors to identify priorities (e.g. examples from different continents: Zhang et al. 2014; Campagnaro et al. 2018; Carvalho et al. 2020). These works can be used to make additional comments on the need of spatial information and compare different proposed tools. These works can also help in highlighting possible integrations to the spatial tool that authors are presenting.

Thank you for your suggestions. However, although it is not the aim of the paper to review different other methods and tools, we have integrated a paragraph giving a short overview in the introduction, provided some ideas for integration of appropriate spatial conservation prioritization approaches in the section "Further development" and referred to other software tools, types of analyses and methodological approaches, which could be used in sophisticated combined approaches in the discussion section.

S1: I think that some examples can be reported in the main text (see comments on the method section). For example, "Reference Area: Asia border" and "Focal Area: Asian countries". This would benefit comprehension of the national responsibility tool.

and

S1 L18-20: I suggest reporting these possible issues in the main text.

We are grateful for these suggestions and have moved the whole content of Supplement S1 to the part "Technical requirements" in the section "Description of the National Responsibility Assessment Tool" of the main text. Additionally, as mentioned above, we have already added some new paragraphs to explain the approach a little bit more in detail with an other example in the "Methods" section.

S2 L12: Small typo: "CNTRY_NAME".

This is not a typo, it is the name of the field for the Focal Region you can see in right panel of the user interface in Figure S2.1. Shapefiles use dBase format for attribute tables. dBase field names are limited to the string length of 10 characters only. Because we have moved the whole content of S1 to the main text S2 is now S1.

S3: Are the reported examples of possible cases already presented in other publications? I find this supplementary extremely useful to better understand possible cases.

Thank you! No, these examples are purely theoretical and have not been published so far. We only used them to better illustrate the difference between both approaches addressed in it. We feel that this artificial construct is more helpful to understand the problems we have to deal with than an example with real maps and species distributions where the reader can be distracted by shape and size of a certain polygon. However, we felt it is better to keep it separated from the main text. Because we have moved the whole content of S1 to the main text S3 is now S2.

Reviewer #2:

Thank you for the opportunity to review this manuscript. This study develops and demonstrates a GIS-based tool, which can be used to produce National Responsibilities and Conservation Priorities for multiple species using freely available data. For me, this study demonstrates a great tool, which is relatively easy to use and has a pretty good flexibility to be applied on different scales and locations. Furthermore, using freely available species distribution and ecoregion data and setting a standardized assessment for countries are the strengths of this tool. In my opinion, this can certainly contribute to setting conservation prioritizations for policy makers.

Although I know the focus of this study is about the tool itself, I have a question about the method behind this tool, which is the issue about wide-ranging nomadic species and migratory species. Could this method cause an issue like "tragedy of the commons" for wide and evenly distributed species? For example, would wide-ranging nomadic species be considered as basic in NR throughout all countries it lives? How would you address this issue in the method?

Thank you for raising this question. Indeed, a wide ranging or migratory species with a distribution area covering many countries and eco-regions would be probably considered rather as basic throughout all countries it lives. That means that no country would have a special National Responsibility. However, in this case the IUCN Red List status would have more weight and the burden should be shared between all countries. It doesn't mean that this species must not be protected, it only means that not one or some more countries would have a higher responsibility because the species mainly occurs in it or them respectively. The scores of National Responsibility will be correlated with the size of the countries in this case. The country with the largest area would also have the highest responsibility.

Especially for migratory species, detailed analyses could be made focusing either only on the breeding range, areas which are either important during the time of migration or in winter. There are a lot of options for such analyses. However, this is not a question of the tool or special options in it, it is rather a question of selection of species, geographical focus, and stratification in the data. Hence it is in the responsibility of the researcher and the design of the study.

To address and answer such questions we have integrated already options to change the parameters for the analyses (Supplement S1, Fig. S1.2b) and the two approaches. However, it takes more special investigations with standardised theoretical and real data as well to find out more about such effects and possible causes of bias. This is planned for the future and also one reason why we want to provide this tool to the community and other researchers to address such problems.

In Runge et al. (2015), they suggest that it might be more suitable to use the minimum range nomads occupy across multiple years because of their fluctuated distribution. The minimum range might contain some climate refugial sites or high quality habitats for those wide-ranging species.

Runge et al. (2015) Geographic range size and extinction risk assessment in nomadic species. Conservation Biology

Thank you. This is an interesting aspect. However, the tool presented is not limited regarding this. It depends on research question, knowledge of the researcher, aim of the study etc., which polygons will be used for the analysis. Our tool only helps to automatize some steps within this work flow it does not answer questions or make decisions the researcher should do.

For migratory species, I'm curious about how you deal with them in the study. Would you mind to provide some information about this in the manuscript? since this study also includes several migratory species. Do you use their breeding ranges, non-breeding ranges, or passage ranges, or all combined, or all but separated (which means one migratory bird might be counted as NR and CP in countries where it breeds, winters, and passes by). I suppose the last method, which is using all ranges separately, make more sense because migratory populations rely on all habitats for different life stages. However, some migratory birds in Asia might have their non-breeding ranges or passage ranges extend to Oceania (e.g., Australia and New Zealand)

This may influence your results (Fig. 4, and 5) because the proportion of migratory birds among all avian communities can be very high in the north hemisphere. See Somveille et al. (2013) Mapping Global Diversity Patterns for Migratory Birds. PLOS ONE.

Thank you for raising this question. Indeed, migratory species need a special treatment and more sophisticated analyses and interpretation of results in such studies. Depending on the question a researcher has, it is possible to use separate breeding, wintering and passing by ranges with the tool, if the researcher separates these data in the input files. However, our example was only meant as an illustration of the opportunities. It was not our aim to do a detailed analysis in this case. This is planned for the future. We only mentioned migratory and widespread species because we didn't want to exclude such species from the analysis. A complete assessment would require the detailed discussion of each species and, therefore, go far beyond the aim of our manuscript.

Minor comments as follows

line 32: to prioritize conservation what? to prioritize conservation resources? funds? effort? Actions?

Thank you! We have specified this in the respective sentence.

line 54: could you provide one or a few sentences in the introduction to define or explain "national responsibility"? If I didn't read any previous studies about NR, I would only realize what it really is until the Methods…

We have added a sentence to explain this concept. Thank you!

line 184: While these two BUs may be useful for terrestrial species, incorporating "Freshwater Ecoregions of the World" might further enlarge the applicability of this tool. The distribution of freshwater biodiversity maybe more relevant to the shape and size and boundary of watersheds/river basins than local climate or terrestrial vegetation. The freshwater ecoregion data is also freely available online. See Abell et al. (2008)

Freshwater ecoregions of the world: A new map of biogeographic units for freshwater biodiversity conservation. BioScience.

Thank you for mentioning this aspect. Indeed, the tool and this approach are not limited to terrestrial ecosystems. We have added a paragraph explaining this and referring to resources for freshwater and marine ecoregions.

line 312: Which part of your results supports this statement about "sensitive"? Is there any quantified sensitivity? such as sensitivity analysis or a plot about the correlation between the range size of FS and biophysical regions and NR? or simply based on the description.

We didn't do any specific sensitivity analysis. However, we got already some experience in former analyses and one also can derive such effects simply from the type of analysis and distribution of polygons. However, as already mentioned before in this letter and also mentioned at the end of the discussion in our manuscript, one aim to develop and release this tool was to support such type of studies. We added additional sentences at the end of the "Discussion" section in the manuscript.

line 528: "probability" or "responsibility"?

Thank you! We have revised to "responsibility"

Additional information

The program will be described in github.com and sourceforge.org with the following text:

ArcGIS-NRA-Tool

ArcGIS Tool for the Assessment of National Responsibilities for Endangered Species and Habitats

The aim of the source code is to develop a National Responsibility Assessment Tool that can be used as plug-in for ArcGIS (https://github.com/popecologist/ArcGIS-NRA-Tool and http://popecologist.github.io respectively or https://sourceforge.net/projects/arcgis-nra-tool/).

There are two binary versions of the add-in: SpeciesTool.NET.20140619.esriAddIn - Version from 2014-06-14 compiled with Visual Studio 12, Microsoft .NET 3.4, and the matching (older) SDKs ArcGISRuntime and ArcObjects from ESRI. SpeciesTool.NET.20200228.esriAddIn - Version from 2020-02-28 compiled with Visual Studio 17, Microsoft .NET 4.6.1, and the and the matching (newer) SDKs ArcGISRuntime 100.2.0 and ArcObjects for ArcGIS 10.6.1 from ESRI.

Copyright (c) 2020 Yu-Pin Lin / 林裕彬 Department of Bioenvironmental Systems Engineering National Taiwan University No. 1, Sec. 4, Roosevelt Road Taipei 10617 Taiwan R.O.C. Office Phone: 886-2-33663467; Fax: 886-2-2368-6980 E-mail: yplin@ntu.edu.tw http://homepage.ntu.edu.tw/~yplin/Scales-Taiwan.htm

Credits: Academia Sinica (programming) Reinhard A. Klenke (revising, updating)

The development of the ArcGIS-NRA-Tool was mainly funded by Ministry of Science and Technology of Taiwan (former National Science Council of Taiwan, code NSC101-2923-I-002-001-MY2), and a contribution from the EU FP7 project SCALES: Securing the Conservation of biodiversity across Administrative Levels and spatial, temporal, and Ecological Scales, under the European Union's Framework Program 7 (Code: 226852 FP7-ENVIRONMENT ENV.2008.2.1.4.4., www. scales-project.net.

This program is free software: you can redistribute it and/or modify it under the terms of the GNU General Public License as published by the Free Software Foundation, version 3. This program is distributed in the hope that it will be useful, but WITHOUT ANY WARRANTY; without even the implied warranty of MERCHANTABILITY or FITNESS FOR A PARTICULAR PURPOSE. See the GNU General Public License for more details.

You should have received a copy of the GNU General Public License along with this program. If not, see http://www.gnu.org/licenses/. Please cite and refer to the tool in any report, scientific paper or other type of publication either in print or electronically with the reference mentioned below.

Unfortunately we still need proprietary libraries from third parties such as ESRI (e.g. ArcGIS SDK, ArcObjects SDK) and from Microsoft to be linked again this source code. The need for these publicly available but proprietary libraries is a drawback. Therefore, we suggest to anyone who thinks of doing substantial further work on the program to give highest priority to tasks changing the program in a way that it can do the same job without the proprietary libraries.

Installation of the ArcGIS-add-in: There might be a "No GUI components found in this Add-In. Add-In version does not match." error during installation. This error only occurs when a user tries to install the add-in using the in the Customize dialog. When the add-in is double-clicked from Windows Explorer, the add-in is installed successfully. This is a defect (NIM095435 http://support.esri.com/bugs/nimbus/TklNMDk1NDM1. A potential workaround is to either: the Add-In in Windows Explorer to install OR add folder through in Add-In Manager (see more at: https://community.esri.com/thread/162324).

References: Lin Y-P, Schmeller D S, Ding T S, Wang Y Ch, Lien W-Y, Henle K, Klenke R A (2020): A GIS-based policy support tool to determine national responsibilities and priorities for biodiversity conservation. PLOS ONE. doi: xxxxxx

Each separate file of the C# code is starting with the following lines referring to the GNU General Public License and to the Article in PLOS ONE:

```
/*
 * This file is part of the ArcGIS National Responsibility Assessment Tool
 * source code (https://github.com/popecologist/ArcGIS-NRA Tool or
http://xxx.github.io).
 *
 * Copyright (c) 2020 Yu-Pin Lin / 林裕彬
 * Department of Bioenvironmental Systems Engineering
 * National Taiwan University
 * No. 1, Sec. 4, Roosevelt Road
 * Taipei
 * 10617 Taiwan
 * R.O.C.
 * Office Phone: 886-2-33663467; Fax: 886-2-2368-6980
 * E-mail: yplin@ntu.edu.tw
 * http://homepage.ntu.edu.tw/~yplin/Scales-Taiwan.htm
 *
 * Credits:
 * Academia Sinica (programming)
 * Reinhard A. Klenke (revising, updating)
 *
 * The development of the ArcGIS-NRA-Tool was mainly funded
 * by Minister of Science and Technology  of Taiwan
 * (National Science Council of Taiwan) (NSC101-2923-I-002-001-MY2),
 * and a contribution from the EU FP7 project
 * SCALES: Securing the Conservation of biodiversity across
 * Administrative Levels and spatial, temporal, and Ecological Scales,
 * under the European Union's Framework Program 7
 * (grant Code: 226852 FP7-ENVIRONMENT ENV.2008.2.1.4.4.;
 * www. scales-project.net.
 *
 * This program is free software: you can redistribute it and/or modify
 * it under the terms of the GNU General Public License as published by
 * the Free Software Foundation, version 3.
 *
 * This program is distributed in the hope that it will be useful, but
 * WITHOUT ANY WARRANTY; without even the implied warranty of
 * MERCHANTABILITY or FITNESS FOR A PARTICULAR PURPOSE. See the GNU
 * General Public License for more details.
 *
 * You should have received a copy of the GNU General Public License
 * along with this program. If not, see <http://www.gnu.org/licenses/>.
 *
 * Please cite and refer to the tool in any report, scientific paper or
 * other type of publication either in print or electronically with the
 * reference mentioned below.
 *
 * References:
 * Lin Y-P, Schmeller D S, Ding T S, Wang Y Ch,  Lien W-Y, Henle K,
 * Klenke R A (2020): A GIS-based policy support tool to determine national
```

```
 * responsibilities and priorities for biodiversity conservation.
 * PLOS ONE. doi: xxxxxx
*/
```
